# When does poor subjective financial position hurt the elderly? Testing the interaction with educational attainment using a national representative longitudinal survey

**DOI:** 10.1186/1471-2458-11-166

**Published:** 2011-03-17

**Authors:** Christy Pu, Nicole Huang, Gao-Jun Tang, Yiing-Jenq Chou

**Affiliations:** 1 Institute of Hospital and Health Care Administration, School of Medicine, National Yang-Ming University, Taipei, Taiwan; 2 National Yang-Ming University Hospital, Yi-Lan, Taiwan; 3 Department of Public Health, School of Medicine, National Yang-Ming University, Taiwan

**Keywords:** aged, education, health, depressive symptoms, mortality

## Abstract

**Background:**

Several studies have demonstrated that perceived financial status has a significant impact on health status among the elderly. However, little is known about whether such a subjective perception interacts with objective socioeconomic status (SES) measures such as education that affect the individual's health.

**Methods:**

This research used data from the Survey of Health and Living Status of the Middle Age and Elderly in Taiwan (SHLS) conducted by the Bureau of Health Promotion, Department of Health in Taiwan. Waves 1996, 1999 and 2003 were used. The sample consisted of 2,387 elderly persons. The interactive effects of self-rated satisfaction with financial position and educational attainment were estimated. Self-rated health (SRH), depressive symptom (measured by CES-D) and mortality were used to measure health outcomes.

**Results:**

Significant interaction effect was found for depressive symptoms. Among those who were dissatisfied with their financial position, those who were illiterate had an odds ratio (OR) of 8.3 (95% CI 4.9 to 14.0) for having depressive symptoms compared with those who were very satisfied with their financial position. The corresponding OR for those with college or above was only 2.7 (95% CI 1.0 to 7.3). No significant interaction effect was found for SRH and mortality.

**Conclusions:**

Although poor financial satisfaction was found to be related to poorer health, the strongest association for this effect was observed among those with low educational attainment, and this is especially true for depressive symptoms. Subjective financial status among the elderly should be explored in conjunction with traditional measures of SES.

## Background

The relationship between socioeconomic (SES) and health disparities has been well documented, with those having lower SES being less healthy. However, it has been found that medical care and health behavior do not sufficiently account for this relationship and the psychosocial stress caused by inequalities is one of the potential explanatory factors [1]. The relationship between SES and health disparities thus can result from two pathways, the material and the psychosocial [2]. While the importance of the former lies in social policies that deal with resource distribution, the latter provides information on the behavioral and psychological responses to these disparities [2]. Recently, there has been an increasing interest in exploring the relationship between subjective SES and health disparities [3-7].This branch of research suggests that ''psychological stress'' plays an important role in affecting health adversely.

Subjective SES consists of a complex measure of a person's relative standing in society, and is often defined as "an individual's perception of his/her place in the socioeconomic structure" (p.1321) [8], which may include both social and economic phenomena. Subjective SES is important in research on health disparities because it has been shown that SES may operate at the social level [9]. It has been argued that a person's relative standing may be more important than his absolute SES in terms of influencing health [10]. In addition, previous research has indicated that distribution of income and neighborhood characteristics are closely associated with health and sometimes even have stronger effects than an individual's SES [11-13]. These inequality effects on health may manifest through subjective SES, for example through mechanisms such as psychological well-being and behavioral choices; importantly, the distress caused by such inequalities may not be captured by objective SES [14].

The above mentioned research on subjective SES suggests that if one wishes to reduce health disparities, then the relationship between subjective SES and health status should not be overlooked. Several studies have found a significant relationship between subjective SES and health (such as self-rated health, coronary heart disease and all cause mortality) [7,15-17]. For the elderly, subjective or perceived SES is of particular importance because traditional SES measures such as income, occupation and education are limited when measuring SES during old age. For older adults, income and occupation may not be relevant due to retirement, and the benefits reflected by education may not be the same as those obtained from other attainments [18].

Subjective SES is a multidimensional concept and many measures have been proposed in previous research. Each of these measures captures a different aspect of what is termed "subjective SES". One of the frequently used measure of subjective SES is a question involves a ten rung ladder asking the respondents to place themselves in the ladder, with the top of the ladder representing those who are best off in the society (who have most money, most education and best jobs) [19]. A modification has been made by asking the respondent to compare themselves with different reference groups (others in the society, others of the same race or ethnicity or neighbors and parents when they were at the same age) [15]. One study used individual perceptions of workplace status (whether the respondents perceive themselves as ''employees'', ''foremen'', or ''managers'') as the measure of subjective SES [17]. Relative comparison (to doctors and to garbage collectors) pertaining to occupational prestige as well as perceived job control have also been examined [16,20].

Within different measures of subjective SES, subjective financial position has been shown to have separate effect on health independent of other SES measures. For example, Butterworth et al[21] has shown that financial hardship is particularly associated with depression independent of the effect of absolute income. Szanton et al. [18] found that women who reported financial strain had a higher risk of death, and that financial strain is a stronger predictor of mortality than annual income among older women. Similarly, Zimmerman and Katon [22] found that employment status and financial strain are causally related to depression, but that income is not. This suggests that how people perceive their financial position may be more important in terms of affecting their health than their actual income level.

Financial position has been measured differently in various studies. Other than income level, some have asked the respondent whether there is any money left over at the end of month [18], others have asked whether they are suffering from financial hardship [21], and yet others have asked whether they have difficulties meeting certain expenses [23]. However, it is possible that individuals with similar income or financial hardship levels have different measures of financial satisfaction due to, for example, expectations, and it may be difficult to determine what reference group the individuals use when trying to match such expectations. In this research, we use financial satisfaction as the measure for subjective financial status. One merit of using *satisfaction *as the measure of subjective financial status is that it (at least partially) mitigates the problem caused by people using different reference groups when assessing their social position. Wolff et al[15] demonstrated that the relationship between subjective SES and SRH may be sensitive to the reference group used when measuring a person's relative standing. A person may have a lower SES rank when a more distal reference group (such as "others in the society") is used, but he may not necessarily be dissatisfied with it since those at the top positions in the society may simply be perceived as unattainable by the majority of the population and hence this does not necessarily lead to distress. In other words, a low rank using a distal reference group may not necessarily mean a low rank when the reference group is narrowed. Using *satisfaction *on the other hand, may reflect more of such distress if it is present.

While many studies have noted that objective and subjective SES in themselves are not mutually exclusive pathways and may be inter-related [2,24], few research studies have specifically tested the interactive effect between the two. Poor health conditions are normally more prevalent in the lower socioeconomic groups, and hence it may be hypothesized that a lower subjective SES (such as poorer perceived financial status) may act as either a mediating factor with respect to the SES-health gradient or interact with the SES variables to produce poor health. This phenomenon has been shown to occur with work stress, where the prevalence of poor health was found to be highest among those with high work stress and poor SES [1]. Similarly, for subjective financial status, it is possible that this measure does not work independently of objective SES measures such as educational attainment when affecting an individual's health. This issue is particularly important for the elderly since it is more difficult to obtain a suitable SES measure for this subgroup, and a complete measure may include both objective and subjective measures of SES. In this study, we explored the hypothesis that there is an interaction between subjective financial satisfaction and education and that this is associated with health outcomes. We tested this association with respect to three health measures, namely self-rated health (SRH), depressive symptoms, and mortality among the elderly in Taiwan. These three health measures have been shown to be related to traditional measures of SES; specifically, individuals with poor SES are more likely to have poorer SRH, higher depressive symptoms and a higher risk of mortality [25-30].

## Methods

### Data

This research uses data from the Survey of Health and Living Status of the Middle Age and Elderly in Taiwan (SHLS) conducted by the Bureau of Health Promotion, Department of Health in Taiwan. The SHLS is a 15-year longitudinal survey based on a national representative sample that was initiated in 1989. In 1989, the SHLS started with 4,049 respondents (response rate = 91.8%) aged 60 and above. The age range of this sample was 60 to 96 years, with those aged between 60 and 80 representing about 95% of the total sample. The SHLS was conducted by the Bureau of Health Promotion under the Department of Health in Taiwan. The sampling frame was designed to ensure that the sample is representative of the elderly in the country. A detailed description of the sampling method can be found elsewhere [31]. Information was collected by face-to-face interview, and the survey consists of a rich dataset on the elderly in Taiwan; it includes demographics, socioeconomics, life style, as well as health status. Starting in 1996, the survey included the CES-D (10 items) questions and hence information on depressive symptoms was available for waves 1996, 1999 and 2003. These three waves were used for the analysis. Since the initiation of the survey (n = 4049), 1,047 subjects had died by 1996 and of the remaining 3002 subjects in 1996, 333 did not complete the survey (response rate = 88.9%). For the remaining 2669 subjects, 282 had at least one missing value for the variables used (as described below), and this left us with 2,387 subjects for analysis. Among the 282 subjects, those with missing values for financial satisfaction tended to have lower education (p < 0.01). This should be taken into consideration when interpreting the results. The descriptions of the variables are given below.

### Socioeconomic measures

#### Education

Educational attainment was used as a measure of objective SES. Since the respondents were the elderly persons, it is illogical to use income or occupation as an objective SES measure since a large proportion of the respondents would be retired. In terms of education variable, the respondents were grouped into "illiterate", "literate but with no formal education", "primary school", "junior high school", "senior high school and "college or above", and this assignment was assumed to be unchanged across the waves, which is be a reasonable assumption for the elderly.

#### Subjective financial status

For this research we use the following. The respondents were asked the question "generally speaking, are you satisfied with your financial status?" and the respondent was able to choose from "Very satisfied", "Satisfied", "Average", "Unsatisfied", "Very unsatisfied". To ensure that there were sufficient respondents within each group, we combine those who responded "Unsatisfied" with those who responded "Very unsatisfied". This is a time-varying variable such that the respondent may report different level of satisfaction at each wave and was treated as such during the various regression analyses.

#### Other control variables

All regressions were controlled for respondent's marital status and ethnicity (Fukien, Hakka, Mainlander, and other). Fukien, Hakka and Mainlander represent the three major ethnicities in Taiwan. Other control variables included whether the subject is a current smoker, and the number of persons living together in a household.

### Health measures

Three health measures were included in the analysis, namely self-rated health (SRH), depression, and mortality. For SRH, each respondent was asked the question " How would you rate your general health?" and the respondent could choose from "very good", "good", "fair", poor" and "very poor". This question was asked for all waves and was treated as time-variant. For each wave, SRH was dichotomized into those who reported reduced health (SRH not equal to "very good" or "good") and those who did not; this approach is consistent with previous research [1,32]. Sensitivity analysis was done using two other cut-off points to categorize those who reported reduced health, namely SRH not equal to "very good" alone and SRH not equal to either "very good", "good" or "average". Depression symptoms were measured using the Center for Epidemiological Studies-Depression scale (CES-D, 10-item) score. The presence of depressive symptom was identified when the score was equal to or greater than 10 [33,34]. Finally, mortality was also used as one of the health outcomes.

### Statistical analysis

Sample characteristics are presented as percentages for categorical variables and as means for continuous variables (Table 1). Chi-square and ANOVA were used to determine significant differences in trends across the waves for the categorical and continuous variables, respectively. Bivariate analysis for SES and each of the three measures of health are presented in Table 2. The generalized estimation equation (GEE) was used to estimate the models for SRH and depressive symptoms, and Cox regression was used to estimate the impact of SES on mortality. The main effects of the three SES measures on each of the health outcome are presented in Table 3. Finally, we estimate the interactive effect between financial satisfaction and the education, which is shown in Table 4. We also performed statistical tests for the interaction effects, as well as the simple effect for financial satisfaction at each level of education.

**Table 1:** Sample characteristics for the survived (mean or % by wave)

Year	1996	1999	2003	*p*^a^
N	2387	2022	1482	**
Age (mean)	73.8+-5.0	76.4+-4.8	79.6+-4.1	
Sex (male, %)	56.1	55.1	54.5	
**Education (%)**				
*Illiterate*	36.0	36.6	34.0	
*Literate with no formal education*	8.4	8.4	8.2	
*Primary school*	34.0	34.0	34.9	
*Junior high school*	9.5	9.5	9.8	
*Senior high school*	6.0	6.0	7.0	
*College or above*	5.5	5.5	6.2	
**Subjective financial status (%)**				**
*Very satisfied*	7.5	6.9	5.7	
*Satisfied*	33.4	32.0	41.4	
*Average*	43.3	40.9	33.5	
*Dissatisfied*	15.9	20.3	19.3	
**Self-rated health**				**
Very good	12.2	9.2	7.9	
Good	19.4	19.4	21.0	
Faire	34.2	34.4	32.1	
Poor	28.9	29.9	32.5	
Very poor	5.4	7.1	6.6	
**CESD-10 score**	6.5+-6.5	11.0+-3.7	10.5+-3.5	**

^a ^Chi-squared (ANOVA)test for count (continuous) variables for difference in trends across the waves, ** = *p *< 0.01

**Table 2:** Distribution of poor self-rated health, CES-D score and mortality by indicators of SES and financial satisfaction

	Poor self-rated health (%)			Depression (mean)			Mortality (%)		
	1996	1999	2003	1996	1999	2003	1996	1999	2003
**Education (%)**									
*Illiterate*	76.8	81.0	78.6	8.4	11.6	11.3	17.75	13.8	24.5
*Literate with no formal education*	68.7	70.9	64.5	6.9	11.1	10.8	12.0	12.9	20.6
*Primary school*	70.0	70.5	70.8	5.6	10.7	10.4	11.26	11.9	23.0
*Junior high school*	55.3	57.7	66.2	4.8	10.3	9.7	8.23	6.8	15.7
*Senior high school*	49.7	53.7	59.2	4.1	10.2	9.6	6.13	11.9	17.2
*College or above*	42.0	55.7	62.0	4.4	10.0	9.1	10.2	9.9	19.6
**Subjective financial status (%)**									
*Very satisfied*	46.1	40.0	47.1	3.8	9.4	9.8	9.4	9.6	15.1
*Satisfied*	58.2	60.4	65.3	4.5	10.1	9.7	10.4	11.3	21.2
*Average*	74.1	77.8	76.7	6.5	11.2	10.8	12.4	12.5	22.8
*Dissatisfied*	85.2	85.9	81.1	12.1	12.4	12.1	13.7	14.3	24.6
Mean CES-D score									

Number of subjects: 1996 = 2387, 1999 = 2022, 2003 = 1482 (decreases due to death or loss during follow-up)

**Table 3:** Main effects of education and financial satisfaction on health.

	SRH		Depressive symptoms		Mortality	
	OR	95 C.I.	OR	95 C.I.	OR	95 C.I.
**Education**						
*Illiterate (base)*						
*Literate with no formal education*	0.7	(0.5 1.0)	0.8	(0.6 1.0)	0.8	(0.6 1.1)
*Primary school*	0.8	(0.6 1.0)	0.7	(0.6 0.9)	0.9	(0.7 1.0)
*Junior high school*	0.6	(0.5 0.8)	0.7	(0.5 0.8)	0.6	(0.4 0.9)
*Senior high school*	0.5	(0.3 0.6)	0.7	(0.5 0.9)	0.8	(0.6 1.2)
*College or above*	0.5	(0.3 0.7)	0.6	(0.4 0.8)	0.8	(0.6 1.2)
**Financial satisfaction**						
*Very satisfied (base)*						
*Satisfied*	2.2	(1.7 2.8)	1.1	(0.9 1.4)	1.4	(1.0 2.0)
*Average*	4.0	(3.1 5.2)	1.7	(1.3 2.1)	1.5	(1.0 2.1)
*Dissatisfied*	5.3	(3.8 7.2)	4.2	(3.2 5.4)	1.6	(1.1 2.3)

All models were adjusted for baseline age, sex, marital status, ethnicity, smoking, and number of people living together.

**Table 4:** Interaction between education and financial satisfaction^a^

		SRH			Depression			Mortality		
		OR	95 C.I.	*p*	OR	95 C.I.	*p*	HR	95 C.I.	*p*
Education*FS										
Education	Financial satisfaction									
College or above	*Very satisfied (1.1)*	1.0		*	1.0			1.0		
	*Satisfied (2.6)*	4.2	(2.0 8.8)		1.7	(0.8 3.8)		1.1	(0.4 2.9)	
	*Average(1.5)*	4.9	(2.2 10.9)		1.9	(0.8 4.4)		1.0	(0.3 3.1)	
	*Dissatisfied(0.6)*	6.4	(2.4 17.1)		2.7	(1.0 7.3)		1.5	(0.5 5.0)	

Senior high school	*Very satisfied (0.9)*	1.0		*	1.0			1.0		
	*Satisfied (2.8)*	1.8	(1.0 3.4)		1.3	(0.6 2.6)		2.1	(0.6 7.2)	
	*Average(1.7)*	2.5	(1.3 5.0)		1.9	(0.9 4.0)		2.2	(0.6 7.7)	
	*Dissatisfied(0.8)*	3.2	(1.4 7.4)		2.4	(1.0 5.7)		1.7	(0.4 7.7)	

Junior high school	*Very satisfied (0.8)*	1.0		**	1.0		*	1.0		
	*Satisfied (4.0)*	1.8	(0.9 3.3)		0.8	(0.4 1.6)		2.2	(0.7 7.2)	
	*Average (3.4)*	3.4	(1.8 6.4)		0.8	(0.4 1.5)		1.4	(0.4 4.8)	
	*Dissatisfied(1.0)*	4.4	(1.9 10.0)		2.1	(0.9 4.5)		1.7	(0.4 7.4)	

Primary school	*Very satisfied (2.3)*	1.0		**	1.0		**	1.0		
	*Satisfied (12.6)*	1.8	(1.2 2.6)		0.8	(0.5 1.2)		1.5	(0.8 2.7)	*
	*Average(13.6)*	3.2	(2.2 4.6)		1.3	(0.9 1.9)		1.7	(0.9 3.1)	
	*Dissatisfied(5.5)*	5.1	(3.2 7.9)		3.3	(2.1 5.0)		2.2	(1.2 4.1)	

Literate with no formal education	*Very satisfied (0.48)*	1.0		**	1.0		**	1.0		
	*Satisfied (2.4)*	1.3	(0.6 3.0)		1.1	(0.5 2.8)		2.6	(0.6 11.4)	
	*Average(3.8)*	3.3	(1.5 7.3)		2.0	(0.9 4.9)		2.0	(0.5 8.6)	
	*Dissatisfied(1.7)*	5.5	(2.2 13.9)		5.2	(2.0 13.1)		3.1	(0.7 13.7)	

Illiterate	*Very satisfied (1.5)*	1.0		**	1.0		**	1.0		
	*Satisfied (10.4)*	1.7	(1.0 2.6)		1.9	(1.1 3.1)		1.0	(0.5 1.8)	
	*Average(15.9)*	3.3	(2.1 5.2)		3.0	(1.8 5.0)		1.1	(0.6 1.9)	
	*Dissatisfied(8.6)*	6.0	(3.6 10.1)		8.3	(4.9 14.0)		1.0	(0.6 1.8)	
Overall test of interaction (p-value)		>0.05			<0.01			>0.05		

^a^All models were adjusted for baseline age, sex, marital status, ethnicity, smoking, and number of people living together. All regressions also include the main effect of education.

^b^Percentage of observations per category.

**p<0.01, *p < 0.05 for tests of simple effects of financial satisfaction at each level of education.

## Results

Table 1 shows the sample characteristics at each wave. The distribution of SRH, CES-D score and mortality relative to the three SES measures are presented in Table 2. A gradient of SES can be observed, such that, for all waves, those with a higher education level had a lower rate of reduced health response, a lower CES-D score and a lower mortality. A similar trend was observed for the financial satisfaction variable.

The results from the regression analyses are presented in Table 3. First, education has a positive effect on all health measures. For the base models (models without interactions), those with higher education (compared with illiterate) had a lower odds ratio for reporting reduced health. Those with a college degree or above, for example, had an odds ratio of 0.5 (*p *< 0.01) for reporting reduced health. A similar effect was observed for depressive symptoms. Compared with those with the lowest level of education, all other groups had lower odds ratios of depressive symptoms, ranging from OR = 0.6 ~ 0.8 (*p *< 0.05). For mortality, again, those in the higher educational level groups had lower odds ratio for mortality compared with those who were illiterate, with a slight U-shaped hazard ratios (HRs) across the groups, although this was only statistically significant for the junior high school group (HR = 0.6, *p *< 0.01).

Turning now to financial satisfaction, the effect of this variable on the health measures was statistically significant for all health measures. For the base models, those who reported poorer financial satisfaction had higher odds ratios for reporting reduced health compared with those who reported "very satisfied" (OR ranging from 2.2~5.3, *p *< 0.01). For all SRH models, the results were robust in terms of the different cut-off points for categorizing those who reported reduced health (data not shown). Similarly, those who reported "average" and "dissatisfied" for financial satisfaction were much more likely to report depressive symptoms (OR = 1.7 and 4.2, *p *< 0.01, respectively) compared with those who reported "very satisfied". A similar pattern was found for mortality, with those who reported poorer financial satisfaction (relative to those reported "very satisfied") having a higher HR for mortality, though only marginal statistical significance was observed.

The interaction between the subjective financial satisfaction and education are reported in Table 4. Statistical tests revealed the presence of interactions between the two SES variables for depressive symptom (p < 0.01). In terms of the effect on financial satisfaction, for SRH, the odds ratios of poor SRH were found to be consistently higher among those characterized as having lower financial satisfaction for all education groups, however, with a slight U-shaped ORs observed along the education groups. Among those who were dissatisfied with their financial position, the highest odds ratios were observed for the highest (OR = 6.4, p < 0.01) and lowest (OR = 6.0, p < 0.01) education categories compared with those in the "very satisfied" group. For depressive symptoms, higher ORs were observed consistently for those who were dissatisfied with their financial position among the lower education groups. For example, compared with those who were "very satisfied" within the highest education group, those who were dissatisfied with their financial position has an OR of 2.7 for having depressive symptoms within the same education group, and is statistically insignificant (p > 0.05). The corresponding OR for those who were illiterate were 8.3 (p < 0.01). Statistical tests also revealed that significant interaction effects were present for depressive symptoms. No significant simple effect for financial satisfaction was observed for mortality.

## Discussion

While the relationship between traditional SES and health has a much longer history, subjective SES has begun to gain ground when explaining health disparities over the past decade. In many cases, the effect of subjective SES persists even after controlling for objective SES[10]. We have demonstrated here that the poorest health effect occurred in the lower financial satisfaction and lower education groups for depressive symptoms. The results from this research suggest that, while subjective financial position may contribute to the SES-health gradient, its effect may be particularly harmful in terms of having higher depressive symptoms among those who have a lower education level.

We found that the interaction effect was only statistically significant for depressive symptoms and not for SRH and mortality. First, it is possible that those in the lower educational rank had fewer resources when coping with the stress from poor financial satisfaction, and this may lead to depressive symptoms for the elderly as financial stress is one of the important factors leading to depression for this group[33]. It has also been found that those with lower education experience lower life satisfaction in general [35], and thus this may enhance the psychological stress associated with lower education. Secondly, it is also possible that what comprises "financial satisfaction" may differ between those with high and low educational attainment, and the factors associated with financial dissatisfaction for the lower educated individuals may be associated with higher depressive symptoms. For example, those with lower education may be dissatisfied with their financial positions due to actual financial hardship, and financial hardship is closely associated with depressive symptoms[21]. Another possible explanation is that there may be different time lags associated with the impact of SES on different health measures. More specifically, the impact of SES on depressive symptoms may be quicker than SRH and mortality. Future studies can test whether this mechanism is true for the elderly.

This research has several merits and limitations. Studies investigating the relationship between subjective financial position and health rarely compared results among different health measures. One of the merits of this research is that we have incorporated three health measures and we found different interactive effects among these different measures. The results of this research should also be viewed in light of a number of limitations. Firstly, the insignificance of the interaction terms for the SRH and mortality model may be due to sample attrition and because those who would/could not complete the survey or those who had missing values were excluded. In addition, if assuming those belonged to lower SES groups had higher probability of missing responses, then underestimation of the negative impact of lower SES may be suspected. Secondly, we could not include various other factors that may cause poor financial satisfaction. For example, the amount of savings that an individual has will be important when determining a person's financial satisfaction, especially during old age.

In contrast to Macleod et al's [17] results, where they found that perceived work-place status was only weakly associated with mortality, we found that a perceived poor financial satisfaction had a (marginal) significant main effect on mortality, but had no interaction effect with education, and this may be caused by the different measures of subjective socioeconomic position for the two studies as well as the difference in the mean age of the samples. In our case, financial satisfaction may be correlated with actual financial resource availability and hence medical care utilization, which is particularly important for the elderly. The interaction effects we found for depressive symptoms in this research are in line with the argument that objective and subjective SES do not involve mutually exclusive pathways and may be interrelated [2,24]. Recently, a number of studies have investigated whether SES disparities in health reduce as people become older [36-39]. In this context, we found that the effect of both SES measures persists in our sample (as measured by the main effects of the SES variables), which is a sample of elderly persons, and this is true, for all three health outcomes.

We found a U-shaped gradient in mortality across education groups, with the lowest HRs observed for the junior high school group. This probably was due to the fact that the majority of the elderly investigated in this study had a low level of education, and those with an educational level higher than junior high school may have had special characteristics that were not taken into account during the analysis (for example, working extremely hard and hence had a slightly poorer health).

Previous research has found depressive symptoms to be associated with self-rated health (15). To test whether having depressive symptom acted as a mediating factor in explaining the relationship between subjective financial status and SRH, we re-estimated the models for SRH including depressive symptom (CES-D score > = 10) as an independent variable (table not shown). The results showed that in both the baseline and interaction models, the ORs for having depressive symptoms turned out to be larger than 1 and statistically significant. In the baseline and interaction models, the ORs for having depressive symptoms on reporting reduced health were 1.54 (*p *< 0.01) and 2.1 (*p *< 0.01), respectively. In both models, the pattern of the relationship between financial satisfaction and SRH remain unchanged. Having depressive symptom thus may partially, though not fully, act as a mediating factor for the relationship between financial satisfaction and SRH.

It should be noted that subjective financial satisfaction captures only a specific part of what is termed "subjective SES", and there are many other aspects of subjective SES that have not been investigated in this research. For example, the previously mentioned ten rung ladder question (asking respondent to place themselves in their social position) may capture very different information from the subjective financial satisfaction variable used in this research.

The financial satisfaction used in this research may be influenced by many factors not explored in this study. A future question thus is what are the factors affecting a person's subjective financial satisfaction and to what degree does this related to the person's actual income level. In addition, it is important to determine the mechanisms through which the subjective financial position of a person affects their health. It should also be noted that financial satisfaction may or may not be directly related to a person's education. Obviously, it is very possible that those with higher education earn more and hence are more likely to be financially satisfied. On the other hand, it is also possible that a person with high education being dissatisfied with his financial position simply due to the fact that he is relatively less well off financially, rather than being actually deprived in any material way. If the assessment of subjective position systematically differs across objective SES measures, than the differences in the distributions of objective SES measures across samples could lead to differences in the impact of subjective assessments.

## Conclusions

Previous research has shown that a subjective perception of an individual's financial position is important to determining health status. However, although poor financial satisfaction was found to be related to poorer health, the strongest association for this effect was observed among those with low educational attainment, and this is particularly significant for depressive symptoms. Subjective financial status among the elderly thus should not be explored separately from traditional measures of SES. Future research can test whether other aspects of subjective SES (apart from subjective financial satisfaction) also interact with objective SES.

## Competing interests

The authors declare that they have no competing interests.

## Authors' contributions

CP was responsible for data analysis and drafted the manuscript, NH and GT designed the study and helped in drafting the manuscript, YC helped in designing the study and finalized the manuscript. All authors read and approved the final manuscript.

## Pre-publication history

The pre-publication history for this paper can be accessed here:

http://www.biomedcentral.com/1471-2458/11/166/prepub
